# In memoriam

**Published:** 2025-09-30

**Authors:** Jagoda Šušković, Blaženka Kos

**Affiliations:** University of Zagreb Faculty of Food Technology and Biotechnology, Department of Biochemical Engineering, Laboratory for Antibiotic, Enzyme, Probiotic and Starter Cultures Technology

## Abstract

**Figure:**
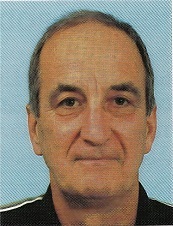

## Prof Dr Sc Srećko Matošić

### (23 January 1939 – 20 July 2025)

On Sunday, 20 July 2025, at the age of 87, our distinguished professor at the University of Zagreb Faculty of Food Technology and Biotechnology, passed away. The funeral service was at Mirogoj Cemetery on 24 July 2025, where we said farewell to our honoured scientist and expert.

Prof Dr Sc Srećko Matošić was born on 23 January 1939, in Split, where he began attending elementary school, finishing it later in Zagreb. He enrolled the Gymnasium in Zagreb, from which he graduated in 1957. In the same year he enrolled at the Department of Chemistry and Technology, Faculty of Engineering, University of Zagreb, and graduated in 1962. He took a job at the Zagreb Paper Factory, where he had a scholarship. The next year he began his academic career at the Department of Biotechnology Faculty of Engineering, University of Zagreb as a scientific assistant on the course Chemistry and technology of antibiotics.

He received his scientific master’s degree in 1971 from the Biotechnology Department of the University of Zagreb Faculty of Technology with the thesis entitled “The influence of selected parameters on the biosynthesis of the antibiotic nisin”. During 1971 and 1972, he completed his specialization at the Istituto Superiore di Sanità in Rome, where he worked on the biosynthesis and isolation of ergot alkaloids. He successfully carried out the research and defended his PhD thesis in the biotechnical field of science, under the title “Contribution to the knowledge of the biosynthesis and isolation of ergot alkaloids from the culture of *Claviceps purpurea* (Fr). Tul.”, at the Faculty of Technology University of Zagreb in 1976.

During his academic career, as assistant professor (1977), he held an important position as Director of the Institute of Food and Biochemical Engineering (1978), and then he was Head of the Food and Biotechnology Department of the Faculty of Technology, University of Zagreb. When he was promoted to the title of associate professor, he was also the first time Head of the Department of Biochemical Engineering. As a full professor, he was elected position of Vice-Dean for Finance at the Faculty of Food and Biotechnology of the University of Zagreb, and another mandate since 2003. He was a full professor with a tenure since 1999.

Expanding its scientific activity, the Laboratory for Chemistry and Technology of Antibiotics was transformed into the Laboratory for Antibiotic and Enzyme Technologies, in 1976, headed by Dr Sc Srećko Matošić as an assistant professor. He remained in that position until 2003, when he was succeeded by Prof Dr Sc Jagoda Šušković and when the scientific and educational activities of the laboratory were expanded again. Namely, due to the widespread and uncontrolled use of antibiotics, as well as the pressing problems with antibiotic resistance, the laboratory established research into the probiotic concept in 1992. Manipulation of the intestinal microbiota with probiotic bacteria to strengthen the body's defence system reduces the risk of infectious diseases, which would result in a reduction in the use of antibiotics, and thus antibiotic resistance spreading. Therefore, new subjects were introduced within the framework of new study programs and the laboratory was renamed into the Laboratory for Antibiotic, Enzyme, Probiotic and Starter Cultures Technology in 2006.

Prof Dr Sc Srećko Matošić had an important influence on the education at the Faculty of Food Technology and Biotechnology at all levels of study programs, from undergraduate study program of biotechnology (Biochemical engineering and Biochemical-microbiology study courses) to postgraduate doctoral study program in Biotechnology and bioprocess engineering, of which he was the long-time leader. He was a course leader of "Antibiotic Technology" and "Enzyme Technology", and since 1998, with the introduction of new study programs, he taught subjects “Biotechnological production of drugs and specific chemicals” and “Enzyme production and enzyme engineering”. He participated in lectures on the subjects: “Process practice”, “Process design”, “Industrial microbiology” and “Biochemical engineering” until 1997, and since then in the subjects “Biotechnological process design”, “Physiology of industrial microorganisms” and “Biochemical engineering I”, and in the postgraduate course subjects: “Biotechnology of industrial antibiotics”, “Development of enzyme production and application process” and “Biochemical engineering”.

He was a guest professor at the Biotechnical Faculty, University of Ljubljana, Slovenia, at the postgraduate study program in Biotechnology, at the course “Biosynthesis of secondary metabolites” from 1992 to 2006.

He was the mentor of numerous bachelor's, master's and PhD theses. During his research career, as a leader of scientific research projects and as co-author of scientific papers, he focused on studies to develop the biotechnological processes of biosynthesis of microbial metabolites (antibiotics, alkaloids, vitamins) and the purification procedures of the microbial metabolites, as well as the production of single-cell proteins on various waste, industrial and agricultural materials, often in collaboration with industrial partners.

Prof Dr Sc Srećko Matošić developed the engineering profession as a co-founder of in Croatian Society for Biotechnology and holder of many positions in this society as well as in Croatian Microbiological Society and Croatian Society of Chemical Engineers. He was also a member of the Croatian Academy of Engineering, to which our laboratory space has been given, so since 2004 the Academy has been headquartered at the address of our former laboratory at 28 Kačićeva Street.

Prof Dr Sc Srećko Matošić will remain in our eternal memory as a colleague, a pleasant collaborator and a mentor, but not only to those of us who worked closely with him. He was a great man who always cherished human values ​​and friendly feelings towards those around him, especially towards his family. He often fondly recalled his childhood spent in Split and Zagreb, and he also often mentioned experiences from hiking trips and happy gatherings with colleagues and friends, many of whom were from his high school and student days.

He was an authority to his students, although as a professor he had a specific casual demeanour. Often during breaks from lectures, he continued to engage his students' attention, but this time with historical short stories, with many remembered details, presenting them with equal authority as he did the engineering topics of his lectures.

His professional and personal contributions will be preserved by his students and colleagues and will not be forgotten.


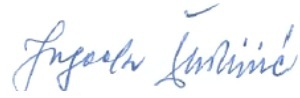
 
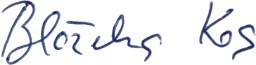


Emerita Prof Dr Sc Jagoda Šušković Prof Dr Sc Blaženka Kos

